# Response to: Comment on ‘The Values and Preferences of People Living With Motor Neurone Disease (MND): A Systematic Review and Meta‐Analysis’

**DOI:** 10.1002/gin2.70089

**Published:** 2026-09-08

**Authors:** Timothy Hugh Barker, Cindy Stern, Camille Schubert, Steve Vucic, Zachary Munn

**Affiliations:** ^1^ Health Evidence Synthesis, Recommendations and Impact (HESRI) School of Public Health, College of Health, Adelaide University Adelaide Australia; ^2^ Adelaide Health Technology Assessment (AHTA) School of Public Health, College of Health, Adelaide University Adelaide Australia; ^3^ Brain and Nerve Research Centre Concord Clinical School, The University of Sydney Sydney Australia

1

We thank Rohtagi et al. [1] for their close reading of our systematic review [2] and for highlighting the importance of synthesising evidence related to values and preferences for informing guideline recommendations. We also thank the authors for raising three main points that we respond to below. We are grateful to the authors for prompting reflection on these issues reported in their letter [1] and for the opportunity to address them via this letter.

## 2 Issue #1: Inclusion of Symptom‐Burden Data

We fundamentally agree with Rohtagi et al. [1] that GRADE defines values and preferences based on the relative importance people place on outcomes [3, 4], and simply rating the current burden of a symptom doesn't constitute a true choice, trade‐off, ranking or utility valuation. In the included study in question [5], participants rated the burden of 10 symptoms from 0 (no problem) to 10 (tremendous problem). As Rohtagi et al. [1] correctly point out, a symptom's burden and its relative importance as a prioritised outcome can diverge.

However, we included these data because GRADE judgements aren't just about identifying which outcomes matter; they are also concerned about the value placed on a change in an outcome. We reasoned that how problematic a patient finds a symptom serves as an, admittedly indirect, indicator of where people living with MND would most value an improvement. The study was eligible under the fourth of our four pre‐specified quantitative study categories, other quantitative methodology assessing outcome importance, which we derived from Zhang et al. [4] and registered a priori [6]. We continue to regard its inclusion as appropriate.

However, from a perspective of prioritising the most critical outcomes or determining the utility of outcomes, we agree with the views of Rohtagi et al. [1]. This could be addressed by rating this information down due to indirectness in our certainty assessments. Three outcomes were informed solely by Raheja et al. [5], eating, mucus and bowel and bladder, and each was rated high certainty without a downgrade for indirectness from the perspective of rating the burden of symptoms. Other groups building on this work may nonetheless make their own decisions regarding whether this represents a form of indirectness that requires downgrading of the certainty of the evidence.

We also noted an error in Supplementary Material 7, where we have mistakenly described the direction of the ALSSQoL response scale in reverse. The extracted values are correct as reported, but the accompanying description should read 0 (no problem) to 10 (tremendous problem). Since these data were synthesised narratively and were never combined with data from any other study, no summary estimate is affected, and no changes are needed in the main document.

## 3 Issue #2: King's Staging Estimates

Rohtagi et al. [1] are correct that our results text and table 1 disagree, and we are grateful this was identified. Table 1 and Figure 2 report stage‐specific floating estimates from a within‐trial interaction model, in which stage contrasts are pooled within each study before being applied to a common reference. This was our primary analysis, and the estimates are printed in the R output provided as Supplementary Material 10 with the published paper [2].

The four values reported in the narrative of Section 3.4.3 were carried over from an earlier version of the analysis in which each stage was pooled as a separate DerSimonian–Laird meta‐analysis, and the text was not updated when the primary analysis was finalised. The sentence in Section 3.4.3 should therefore read: stage 1 (0.71, 95% CI: 0.68–0.74), stage 2 (0.56, 95% CI: 0.54–0.59), stage 3 (0.49, 95% CI: 0.47–0.52) and stage 4 (0.40, 95% CI: 0.36–0.44), with an overall mean of 0.52 (95% CI: 0.44–0.60). These align with the values presented in Table 1 and Figure 2, which are correct as published. Because our certainty ratings and conclusions were based on this primary analysis and not the incorrect text, no conclusions of the review are affected.

## 4 Issue #3: Choice of Meta‐Analytic Model

The Figure 2 caption is accurate. Our decision rule was based on the number of effect estimates entering each model, rather than the number of source publications. Specifically, eight stage‐specific estimates from two studies contributed to the King's model, and 10 contributed to the MiToS model, as such a random‐effects model was used for both. In contrast, the overall EQ‐5D analysis relied on only two estimates, so a fixed‐effect model was used. We acknowledge that our methods text incorrectly refers to ‘studies’ rather than ‘contributing estimates’, and we agree this distinction should have been explicitly explained.

On the sensitivity analysis, the floating‐stage estimates are unchanged by the choice between a fixed‐ and random‐effects specification for the overall model, because they are derived from within‐study contrasts. Only the overall pooled estimate changed, from 0.52 (95% CI: 0.44–0.60) to 0.55 (95% CI: 0.54–0.57) (Supporting Information S1: Material 8). That is what our statement that the two models produced no important difference referred to.

Finally, Rohtagi et al.'s suggestion to stratify the results based on elicitation methods is a reasonable one and supports the argument that we made in the review itself, being methods for eliciting preferences in MND require standardisation, and that a core outcome set would improve the comparability of future evidence. We thank them for their scrutiny, and the clarifications and corrections set out here will be carried into the development of the Australian MND Guideline.

## Author Contributions

**Timothy Hugh Barker:** writing – review and editing. **Cindy Stern:** writing – review and editing. **Camille Schubert:** writing – review and editing. **Steve Vucic:** writing – review and editing. **Zachary Munn:** writing – review and editing.

## Funding

This study was supported by FightMND.

## Conflicts of Interest

The authors declare no conflicts of interest.

## Data Availability

Data sharing is not applicable to this article as no data sets were generated or analysed during the current study.
